# Supplementary material for: MetaQTL: a package of new computational methods for the meta-analysis of QTL mapping experiments
Source: BMC Bioinformatics. 2007 Feb 8;8:49. doi: 10.1186/1471-2105-8-49 (PMC1808479; doi:10.1186/1471-2105-8-49)
Supplement: Additional File 7 — MetaQTL Package : jar file and tutorial. This Zip archive contains both the MetaQTL JAR file and the files of the tutorial. [file 1471-2105-8-49-S7.zip › org.inra.metaqtl/doc/org/metaqtl/bio/util/TabulatedReader.html]

TabulatedReader


|  |  |  |
| --- | --- | --- |
| **Overview** \| **Package** \| **Class** \| **Use** \| **Tree** \| **Deprecated** \| **Index** \| **Help** | |  |
| **PREV CLASS**   **NEXT CLASS** | **FRAMES**    **NO FRAMES**     **All Classes** |  |
| SUMMARY: NESTED \| FIELD \| CONSTR \| METHOD | DETAIL: FIELD \| CONSTR \| METHOD |  |


---


## org.metaqtl.bio.util Class TabulatedReader

```
java.lang.Object
  org.metaqtl.bio.util.TabulatedReader
```

---

``` public final class TabulatedReader extends java.lang.Object ```

This class can be used to parse tabulated file in which the first line
contains the label of the tabulated fields. To use this class is very simple :
  
> ```
>         try {
>                 TabulatedFileReader reader
>                         = new TabulatedFileReader(new FileInputStream("mytabulatedfile.tab"));
>     
>    
>   
>    
>
>  
>   
>    
>     
>           ArrayList keys = tab.getKeys();
>           ... do something with keys 
>                 while (tab.hasNext()) {
>             // new line
>             String value = tab.get("key");
>                   ...    
>                 }
>         } catch (IOException) {
>                 ...
>           }
>         ...
> ```

**Author:**
:   Jean-Baptiste

---

**Constructor Summary**

`TabulatedReader(java.io.InputStream stream)`

`TabulatedReader(java.io.Reader reader)`


| **Method Summary** | |
| --- | --- |
| `void` | `close()`             Closes the stream. |
| `boolean` | `containsKey(java.lang.String string)`             Returns true if the given string is a key of the tabulated file. |
| `java.lang.String` | `get(java.lang.String key)`             Returns the value of the field which belongs to the given key. |
| `java.util.Set` | `getKeys()`             Returns the set of the keys. |
| `java.lang.String` | `getToken()` |
| `boolean` | `hasNext()`             Returns true if there is still a line to read. |
| `void` | `setToken(java.lang.String token)` |
| `int` | `size()`             Returns the number of keys, i.e the number of columns in the file. |

**Methods inherited from class java.lang.Object**

`clone, equals, finalize, getClass, hashCode, notify, notifyAll, toString, wait, wait, wait`

**Constructor Detail**

### TabulatedReader

```
public TabulatedReader(java.io.Reader reader)
                throws java.io.IOException
```

**Throws:**: `java.io.IOException`

---


### TabulatedReader

```
public TabulatedReader(java.io.InputStream stream)
                throws java.io.IOException
```

**Throws:**: `java.io.IOException`


**Method Detail**

### containsKey

```
public boolean containsKey(java.lang.String string)
```

:   Returns true if the given string is a key of the tabulated file. False
    otherwise.

    :   **Parameters:**: `string` - **Returns:**

---


### size

```
public int size()
```

:   Returns the number of keys, i.e the number of columns in the file.

    :   **Returns:**

---


### getKeys

```
public java.util.Set getKeys()
```

:   Returns the set of the keys.

    :   **Returns:**

---


### hasNext

```
public boolean hasNext()
                throws java.io.IOException
```

:   Returns true if there is still a line to read. False otherwise.

    :   **Returns:**: **Throws:**: `java.io.IOException`

---


### get

```
public java.lang.String get(java.lang.String key)
```

:   Returns the value of the field which belongs to the given key.

    :   **Parameters:**: `key` - **Returns:**

---


### close

```
public void close()
           throws java.io.IOException
```

:   Closes the stream.

    :   **Throws:**: `java.io.IOException`

---


### getToken

```
public java.lang.String getToken()
```

:   **Returns:**: Returns the token.

---


### setToken

```
public void setToken(java.lang.String token)
```

:   **Parameters:**: `token` - The token to set.


---


|  |  |  |
| --- | --- | --- |
| **Overview** \| **Package** \| **Class** \| **Use** \| **Tree** \| **Deprecated** \| **Index** \| **Help** | |  |
| **PREV CLASS**   **NEXT CLASS** | **FRAMES**    **NO FRAMES**     **All Classes** |  |
| SUMMARY: NESTED \| FIELD \| CONSTR \| METHOD | DETAIL: FIELD \| CONSTR \| METHOD |  |


---
